# Hydroxychloroquine and azithromycin alter the contractility of living porcine heart slices

**DOI:** 10.3389/fphar.2023.1127388

**Published:** 2023-05-05

**Authors:** Qin Wu, Abigail J. Ross, Tugce Ipek, Georgina H. Thompson, Robert D. Johnson, Changhao Wu, Patrizia Camelliti

**Affiliations:** ^1^ School of Medicine, Jiangsu Vocational College of Medicine, Yancheng, China; ^2^ School of Biosciences and Medicine, University of Surrey, Guildford, United Kingdom

**Keywords:** myocardial slices, organotypic *ex-vivo* models, COVID-19, cardiotoxicity, safety pharmacology, calcium channels, Bay K8644

## Abstract

The cardiotoxicity risk of hydroxychloroquine (HCQ) and azithromycin (AZM) has been the subject of intensive research triggered by safety concerns in COVID-19 patients. HCQ and AZM have been associated with QT interval prolongation and drug-induced arrhythmias, however other cardiotoxicity mechanisms remain largely unexplored. Our group has pioneered the living heart slice preparation, an *ex-vivo* platform that maintains native cardiac tissue architecture and physiological electrical and contractile properties. Here, we evaluated the cardiotoxic effect of HCQ and AZM applied alone or in combination on cardiac contractility by measuring contractile force and contraction kinetics in heart slices prepared from porcine hearts. Our results show that clinically relevant concentrations of HCQ monotherapy (1–10 µM) reduced contractile force and contraction kinetics in porcine slices in a dose-dependent manner. However, AZM monotherapy decreased contractile force and contraction kinetics only at higher concentrations (30 µM). Combination of HCQ and AZM induced a dose-dependent effect similar to HCQ alone. Furthermore, pre-treating porcine heart slices with the L-type calcium channel agonist Bay K8644 prevented the effect of both drugs, while administration of Bay K8644 after drugs interventions largely reversed the effects, suggesting a mechanism involving inhibition of L-type calcium channels. These findings indicate that HCQ and AZM alter cardiac function beyond QT prolongation with significant contractile dysfunction in intact cardiac tissue. Our porcine heart slices provide a powerful platform to investigate mechanisms of drug cardiotoxicity.

## 1 Introduction

Hydroxychloroquine (HCQ), an analogue of chloroquine, is an anti-parasitic and immunosuppressant treating malaria, rheumatoid arthritis, and systemic lupus erythematosus (White, 2007). Despite its safety at concentrations used for the treatment of the above conditions, the recent application of HCQ alone or in combination with azithromycin (AZM) in coronavirus disease-2019 (COVID-19) therapy has raised clinical safety concerns. Higher doses of HCQ used in COVID-19 patients (an order of magnitude higher than the standard dose for malaria) coupled with pre-existing cardiac conditions increase the risk of cardiovascular side effects. Several clinical studies have found that administration of HCQ with or without AZM can result in cardiotoxicity, primarily QT interval prolongation leading to cardiac arrhythmia in some hospitalized patients (Cavalcanti et al., 2020; Chorin et al., 2020; Mercuro et al., 2020; Saleh et al., 2020). Mechanisms underlying HCQ and AZM QT interval prolongation and proarrhythmic risk continue to be the subject of intense basic research. Prolongation in action potential or field potential duration have been reported in Langendorff-perfused guinea-pig hearts (Uzelac et al., 2020; Wang et al., 2021) and human stem cell-derived cardiomyocytes (Kim et al., 2020; Wong et al., 2021). Functional effects on ion channels have been investigated in HEK293 expressing specific human ion channels (Wang et al., 2021) and enzymatically isolated adult human ventricular cardiomyocytes (Jordaan et al., 2021). However, other potential mechanisms of HCQ and AZM cardiotoxicity remain poorly defined.

Recent studies have shown that numerous pharmacological agents alter cardiac function beyond QT prolongation, and can affect contractility, mitochondrial function, cardiomyocyte survival and fibrosis (Mamoshina et al., 2021). Adverse effects of HCQ and AZM on contractility have been reported in human single cardiomyocytes and pluripotent stem cell-derived cardiac tissue constructs (Jordaan et al., 2021; Wong et al., 2021). Although these systems can yield valuable data, they do not replicate the multicellular nature and physiological properties of native cardiac tissue which are critical determinants of both cardiac contractility and drugs responses.

Heart slices are ultrathin sections of living ventricular myocardium, which retain native tissue architecture, including cell types, extracellular matrix, and cell-cell connections, in addition to physiological electrical and contractile properties (de Boer et al., 2009; Camelliti et al., 2011; Perbellini and Thum, 2020). Our group has previously optimised protocols to obtain highly viable slices from the heart of rodent models and large mammals, including human biopsies, which maintain physiological calcium handling, action potential duration, conduction velocity, and robust contractile function (Bussek et al., 2009; Camelliti et al., 2011; Kang et al., 2016; Wen et al., 2018; He et al., 2019). Slices are a powerful platform to assess the therapeutic efficiency and drug-related cardiotoxicity of compounds (Bussek et al., 2009; Camelliti et al., 2011; Himmel et al., 2012; Kang et al., 2016; Miller et al., 2020). Slices prepared from porcine hearts share the anatomical and physiological properties of the human heart but are more readily available. They offer a novel, human-relevant platform and a suitable preclinical model to screen the cardiac safety of therapeutic drugs, bridging the translational gap between preclinical cardiac safety data of potentially cardiotoxic drugs and human patients. In this study, we use the porcine heart slice model to investigate the effect of therapeutically relevant concentrations of HCQ and AZM on cardiac contractility. Specifically, we determine the concentration-dependent effect of HCQ and AZM individually and in combination on the contractile force and contraction kinetics of slices and explore potential underlying mechanisms of action.

## 2 Material and methods

### 2.1 Drugs

Hydroxychloroquine (HCQ; CAS NO. 747-36-4) and azithromycin (AZM; CAS NO. 83905-0-5) were purchased from Cayman Chemicals (Cayman Chemical Company, USA). Drugs were initially formulated in phosphate-buffered saline as a stock solution and diluted to working concentrations in Tyrode’s solution (NaCl 140 mM; KCl 4.5 mM; glucose 10 mM; HEPES 10 mM; MgCl_2_ 1 mM; CaCl_2_ 1.8 mM; pH 7.4). Working drug concentrations within therapeutic range were selected based on previously defined literature (Gielen et al., 2010; de Olano et al., 2019; Wang et al., 2020). (±)-Bay K8644 (BK, CAS No 71145-03-4) was purchased from Merck (Sigma-Aldrich, UK). Stock solution was prepared in DMSO and working concentrations in Tyrode’s solution (0.01% DMSO in final working concentration).

### 2.2 Tissue collection

Porcine hearts were acquired from The Pirbright Institute, UK. Animal experiments were performed under the Home Office Animals (Scientific Procedures) Act (1986) (ASPA) and approved by the Animal Welfare and Ethical Review Board (AWERB) of The Pirbright Institute. Pigs of six to eight weeks of age (male) were housed in agreement with the Code of Practice for the Housing and Care of Animals Bred and were subsequently euthanised with an overdose of 10 mL pentobarbital (Dolethal 200 mg/mL solution for injection, Vetoquinol UK Ltd., Towcester, UK). The procedures were all performed by Personal License holders. Hearts were rapidly excised and perfused with ice-cold cardioplegia solution (NaCl 110 mM; CaCl_2_ 1.2 mM; KCl 16 mM; MgCl_2_ 16 mM; NaHCO_3_ 10 mM; pH 7.4) and transported to the laboratory submerged in ice-cold cardioplegia (transport time <40 min).

### 2.3 Preparation of porcine heart slices

Slices were prepared as previously described (Camelliti et al., 2011; Kang et al., 2016). Briefly, a 1.5 cm^2^ tissue block was cut from the wall of the left ventricle and mounted epicardium down onto the specimen holder of a high precision vibrating microtome (Leica VT1200, UK) using Histoacryl Blue tissue glue. The holder was submerged in 4°C oxygenated (99.5% O_2_) Tyrode’s solution (NaCl 140 mM; KCl 6 mM; glucose 10 mM; HEPES 10 mM; MgCl_2_ 1 mM; CaCl_2_ 1.8 mM; pH 7.4) containing 10 mM BDM. A ceramic blade, set to the 18° setting, was used to cut 300 µm thick slices in the plane of the epicardial surface (tangential plane), at an advancement speed of 0.04 mm/s, vibration frequency of 85 Hz and amplitude 1.5 mm. Slices were gently removed from the microtome with a plastic Pasteur pipette, secured in a netted well under the weight of a stainless-steel ring and submerged in oxygenated Tyrode’s solution containing 10 mM BDM at room temperature until functional assessment. Contractility measurements were conducted in BDM-free oxygenated Tyrode’s solution (NaCl 140 mM; KCl 4.5 mM; glucose 10 mM; HEPES 10 mM; MgCl_2_ 1 mM; CaCl_2_ 1.8 mM; pH 7.4) at 37°C.

### 2.4 Contractility assessment

Contractility measurements were carried out in a horizontal organ bath (Mayflower, Hugo Sachs Elektronik (HSE), Germany) equipped with an F30 isometric force transducer (HSE). The Transducer Amplifier Module (TAM-A; HSE) was used to amplify signals and a PowerLab 8/35 hardware device (AD Instruments) was used for data acquisition. Slices with well aligned muscle fibers were bound to small sterile plastic transparency triangles (6.2 mm wide, 0.1 mm thick) with Histoacryl Blue tissue glue perpendicularly to muscle fibers. Slices were washed in BDM-free oxygenated Tyrode’s solution (NaCl 140 mM; KCl 4.5 mM; glucose 10 mM; HEPES 10 mM; MgCl_2_ 1 mM; CaCl_2_ 1.8 mM; pH 7.4) over 15 min prior to being transferred to the organ bath. In the organ bath slices were attached with minimum preload to the force transducer hook and to the fixed hook and were submerged in 4 mL prewarmed (37°C) BDM-free oxygenated Tyrode’s solution. Slices were continuously superfused with 37°C BDM-free oxygenated Tyrode’s solution and field stimulated at 1 Hz pacing frequency, with a biphasic pulse of 5 ms duration, using a pair of platinum electrodes placed on opposite sides of the bath and connected to a Grass S88 stimulator (Natus Medical Inc., Pleasanton, United States). For determination of preload dependence of contraction force, slices were stretched in steps of 5% every 5 min, with force recorded for 30 s, until isometric contraction reached the maximum before clearly declining. For drug interventions, slices were stretched and left to stabilise for at least 10 min prior to interventions. Subsequently, drugs were administered at increasing concentrations for a 5 min period each, in a cumulative fashion. Analysis of contractility traces was performed offline following experiments using LabChart v7.3.8 software (AD Instruments). For each condition, the last five traces were selected, and amplitude and kinetics were measured. Amplitude was converted to force (mN), using a scale factor of 10 (measured during force transducer calibration). Force was normalised to the cross-sectional area of the heart slice (mN/mm^2^). To assess contractility kinetics, maximum slope of contraction and relaxation were determined in LabChart.

### 2.5 Data analysis

Student t-test was used to compare values from two groups. One-way ANOVA was used to compare values from more than two groups, with significant group difference compared using Tukey’s multiple comparison *post hoc* test. Two-way ANOVA was used when two variables were present. Statistical analysis was performed using GraphPad Prism version 8.4.3 for Windows (GraphPad Software, San Diego, California United States, www.graphpad.com). Data are presented as mean ± SEM, and a *p*-value of <0.05 was considered significant.

## 3 Results

### 3.1 Porcine heart slices generate physiological contractile force

**FIGURE 1:**
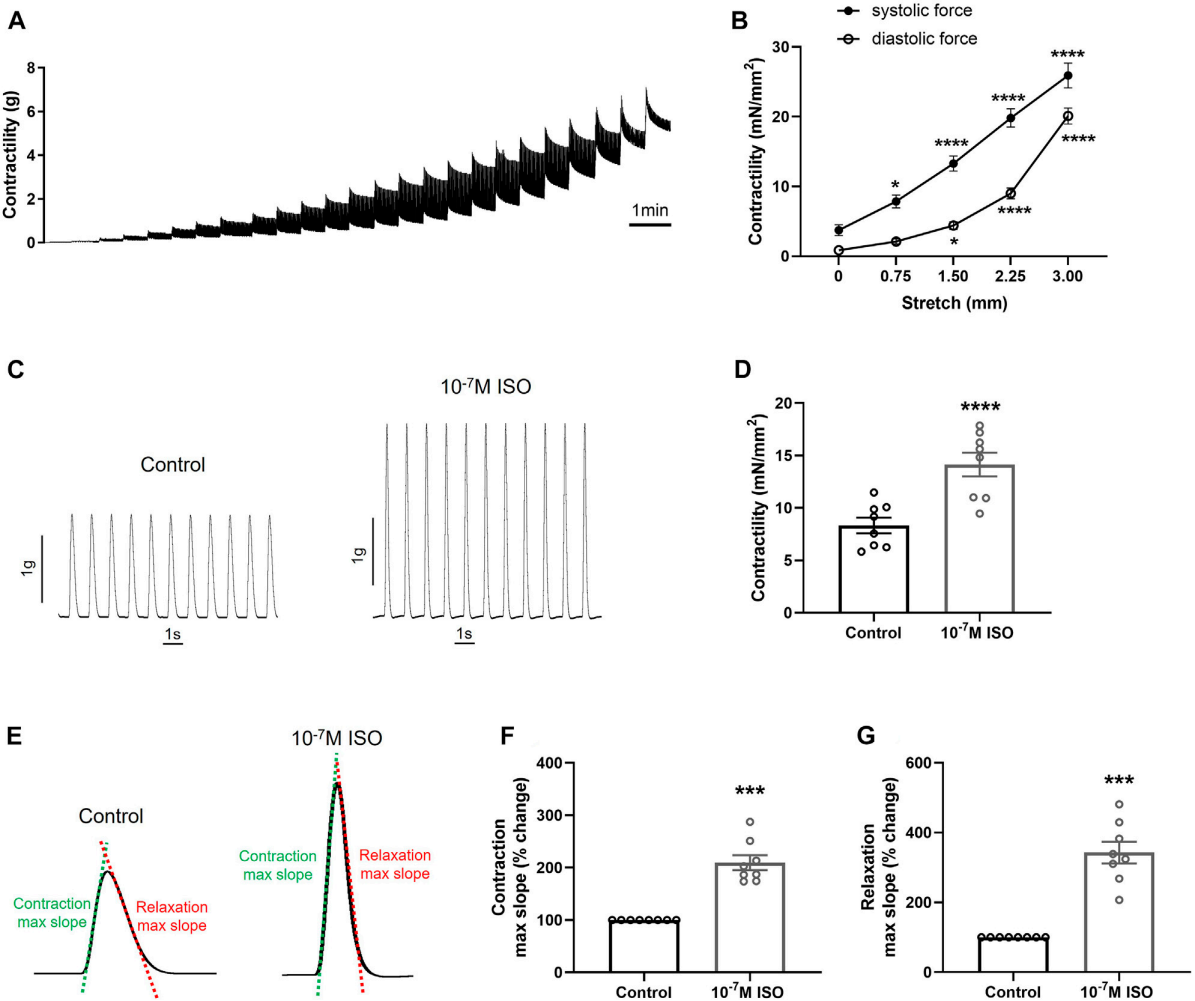
Contractile force generated by porcine heart slices. **(A)** Representative contraction traces from porcine slice stretched in a stepwise manner to maximum force generation. **(B)** Systolic and diastolic forces produced by porcine slices under stepwise stretch and 1 Hz stimulation (*n* = 9 slices/4 hearts). *p < 0.05, ****p < 0.0001 vs. 0 mm stretch (two-way ANOVA with Dunnett’s multiple comparison test). **(C)** Representative contraction traces in response to β-adrenergic stimulation (10^−7^ M isoproterenol—ISO). **(D)** Contractile force in the presence or absence of ISO (*n* = 8 slices/4 hearts). ****p < 0.0001 vs. control (paired *t*-test). Bars represent means ± SEM. **(E–G)** Maximum slope of contraction and relaxation in the presence or absence of ISO (*n* = 8 slices/4 hearts). ***p < 0.001 vs. control (paired *t*-test). Bars represent means ± SEM.

To assess the suitability of porcine heart slices as a platform to study the effect of drugs on cardiac contractility, we characterized the contractile properties of the slices and measured their response to isoproterenol treatment. Using a force transducer and a stepwise stretch protocol, slices were stretched until maximum isometric contraction was recorded at 1 Hz stimulation. As shown in Figure 1, contractility of the slices (force normalized to slice cross-sectional area) increased in a stretch-dependent manner (Figures 1A, B), reaching a maximum value of 10.82 ± 1.09 mN/mm^2^. Importantly, physiological inotropic response to β-adrenergic stimulation was also displayed, with 10^−7^ M isoproterenol increasing contractility by 72% ± 9% (Figures 1C, D). As expected, isoproterenol also increased contraction and relaxation slopes (Figures 1E–G).

### 3.2 Effect of hydroxychloroquine on contractility of porcine heart slices

**FIGURE 2:**
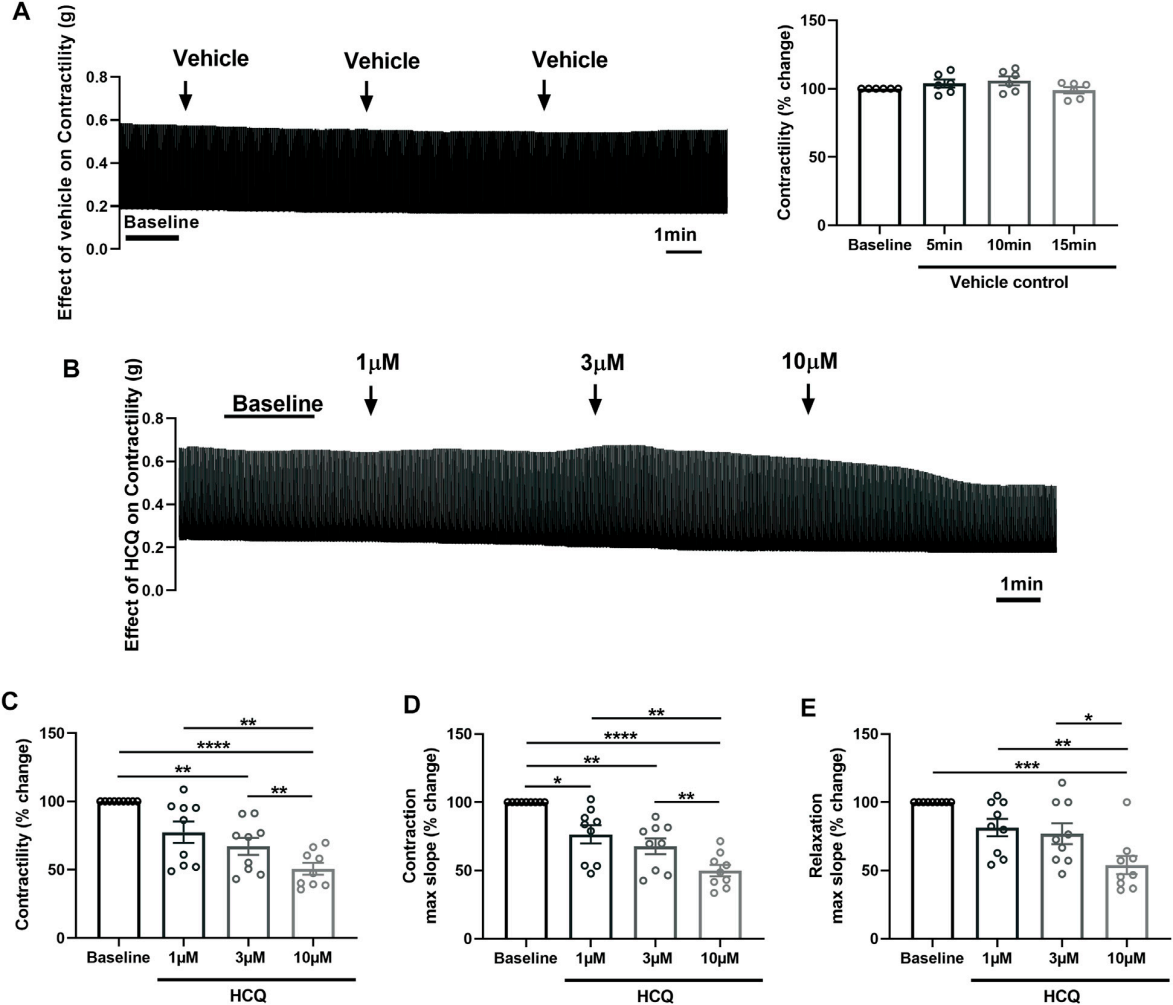
Effect of hydroxychloroquine (HCQ) on the contractility of porcine heart slices. **(A)** Stability of contractility recordings over experimental time in the presence of vehicle control. Left: representative contraction traces. Right: averaged data (*n* = 6 slices/4 hearts; means ± SEM). **(B)** Representative contraction traces from porcine slice in the presence of vehicle control (baseline) and after exposure to increasing concentrations of HCQ (1, 3, and 10 μM). **(C)** Bar graph showing dose-dependent effect of HCQ on contractile force of porcine heart slices (*n* = 9 slices/4 hearts; means ± SEM). **(D,E)** Bar graphs showing dose-dependent effect of HCQ on maximum slope of contraction and relaxation (*n* = 9 slices/4 hearts; means ± SEM). *p < 0.05, **p < 0.01, ***p < 0.001, ****p < 0.0001 (one-way ANOVA with Tukey’s multiple comparisons test).

After confirming viability, maintenance of physiological contractile force and response to β-adrenergic stimulation, we employed porcine heart slices to investigate the effect of HCQ and AZM, administered alone or in combination, on cardiac contractility. Slices were electrically stimulated at 1 Hz and stretched to generate stable baseline contractility in the range of 2–5 mN/mm^2^ (avoiding mechanical tissue damage) before treatment with drugs at clinically relevant concentrations (Gielen et al., 2010; de Olano et al., 2019; Wang et al., 2020; Wong et al., 2021). Time-matched control data obtained using 3 additions of vehicle solution confirmed contractility of slices remained stable over the duration of the experiment (Figure 2A).

Figure 2 shows the effect of increasing concentrations of HCQ (1 μM, 3 μM, and 10 μM), administered cumulatively, on the contractility of porcine slices. HCQ reduced contractile force in a concentration-dependent fashion, with 3 μM and 10 μM significantly reducing force to 68% ± 6% and 51% ± 4% of baseline, respectively (Figures 2B, C). Furthermore, 10 μM HCQ had a significantly larger effect in comparison to 1 μM HCQ and 3 μM HCQ (Figure 2C). Similarly, the maximum slope of contraction was significantly decreased at all HCQ concentrations, with the highest effect observed at 10 μM (Figure 2D). However maximum slope of relaxation was significantly decreased only at 10 μM HCQ (Figure 2E).

### 3.3 Effect of azithromycin on contractility of porcine heart slices

**FIGURE 3:**
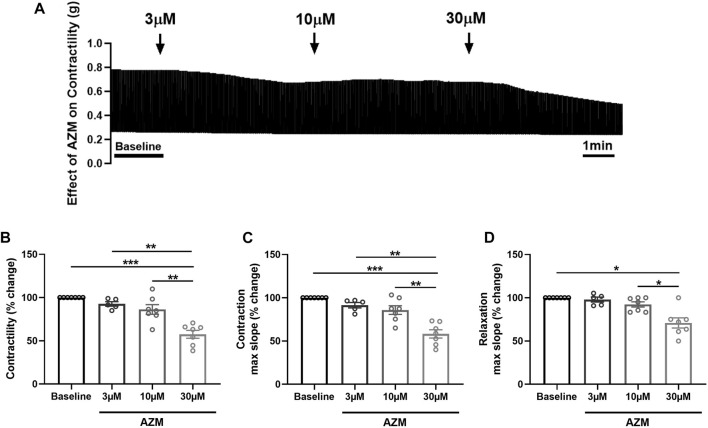
Effect of azithromycin (AZM) on the contractility of porcine heart slices. **(A)** Representative contraction traces in the presence of vehicle control (baseline) and after exposure to increasing concentrations of AZM (3, 10, and 30 μM). **(B)** Bar graph showing the average effect of AZM on contractile force of porcine heart slices (*n* = 5–7 slices/4 hearts; means ± SEM). **(C,D)** Bar graphs showing effect of AZM on maximum slope of contraction and relaxation (*n* = 5–7 slices/4 hearts; means ± SEM). *p < 0.05, **p < 0.01, ***p < 0.001 (one-way ANOVA with Tukey’s multiple comparisons test).

To assess the effect of AZM on cardiac contractility, we treated porcine heart slices with increasing concentrations of AZM (3 μM, 10 μM, and 30 μM), administered in a cumulative fashion. Figure 3A shows typical contraction traces at baseline and after exposure to AZM. AZM did not significantly affect contractile force of slices at 3 μM and 10 μM concentrations, but significantly reduced force to 58% ± 5% of baseline at 30 μM concentration (Figure 3B). Similarly, 30 μM AZM significantly decreased both maximum slope of contraction and relaxation, with 3 μM and 10 μM AZM displaying no significant effect (Figures 3C, D).

### 3.4 Effect of hydroxychloroquine and azithromycin combination on contractility of porcine heart slices

**FIGURE 4:**
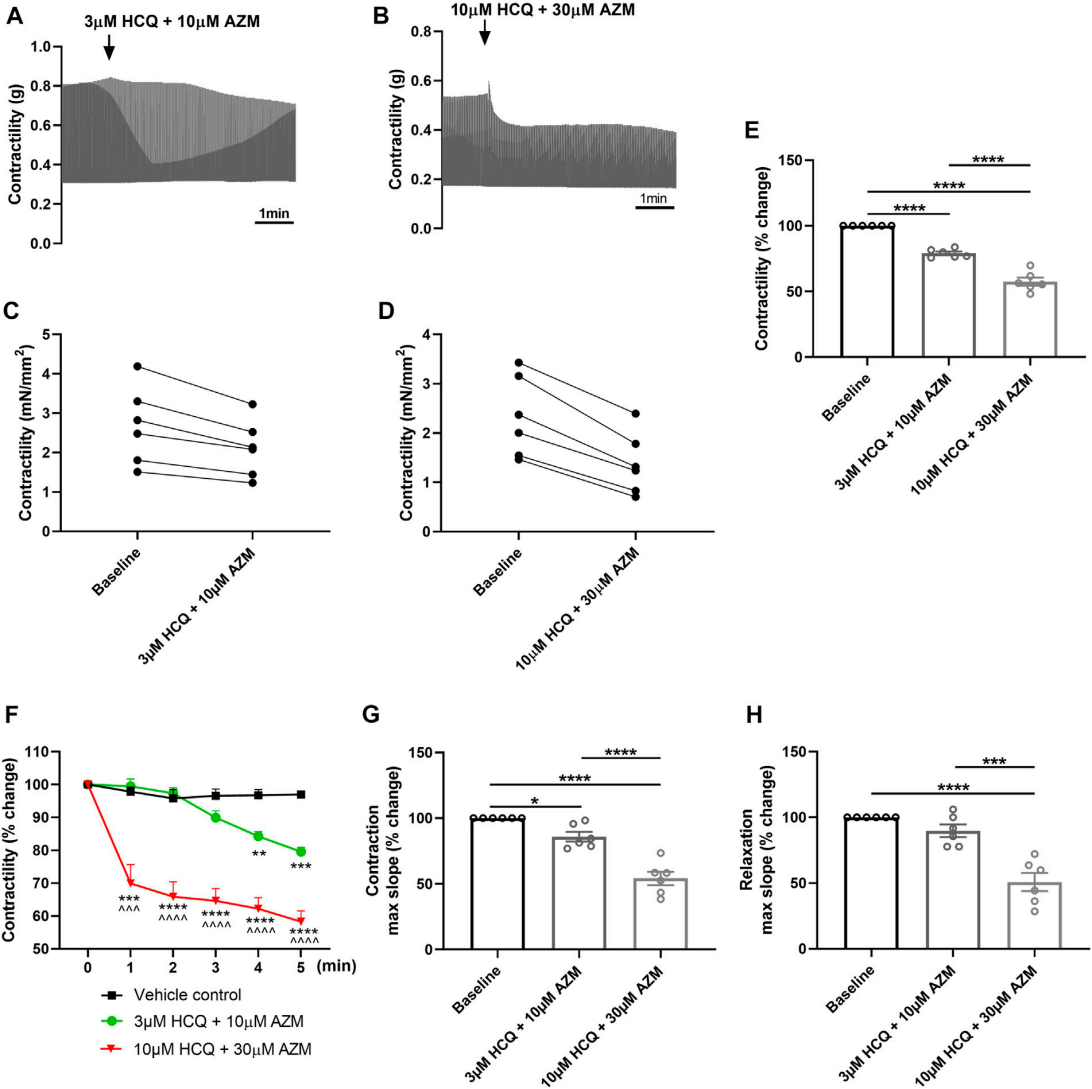
Effect of HCQ and AZM administered in combination on contractility of porcine heart slices. **(A,B)** Representative contraction traces from porcine slices treated with 3 μM HCQ + 10 μM AZM **(A)** or 10 μM HCQ + 30 μM AZM **(B)**. **(C,D)** Paired dot plots showing changes in contractile force from baseline for each individual slice treated with HCQ and AZM combination (*n* = 6 slices/4 hearts). **(E)** Bar graph showing the average effect of 3 μM HCQ + 10 μM AZM and 10 μM HCQ + 30 μM AZM on contractile force of porcine heart slices (*n* = 6 slices/4 hearts; means ± SEM). ****p < 0.0001 (one-way ANOVA with Tukey’s multiple comparisons test). **(F)** Time-dependent effect of 3 μM HCQ + 10 μM AZM and 10 μM HCQ + 30 μM AZM on contractility over the first 5 min exposure (*n* = 6 slices/4 hearts; means ± SEM). **p < 0.01, ***p < 0.001 and ****p < 0.0001 versus vehicle control. ^^^*p* < 0.001 and  ^^^^*p*< 0.0001 versus 3 μM HCQ + 10 μM AZM (two-way ANOVA with Tukey’s multiple comparisons test). **(G,H)** Bar graphs showing effect of HCQ and AZM combination on maximum slope of contraction and relaxation (*n* = 6 slices/4 hearts; means ± SEM). *p < 0.05, ***p < 0.001, ****p < 0.0001 (one-way ANOVA with Tukey’s multiple comparisons test).

As HCQ has been combined with AZM in the treatment of COVID-19 patients (Chorin et al., 2020), we further investigated the effect of HCQ and AZM combination on the contractility of porcine heart slices. Figures 4A, B show typical contraction traces at baseline and after exposure to 3 μM HCQ + 10 μM AZM or 10 μM HCQ + 30 μM AZM, respectively. Figures 4C, D show the response of individual slices to HCQ and AZM combination. Combination therapy of HCQ and AZM significantly reduced contractile force of slices in a concentration-dependent fashion, with 3 μM HCQ +10 μM AZM reducing force to 79% ± 1% of baseline and 10 μM HCQ +30 μM AZM reducing force to 58% ± 3% of baseline (Figure 4E). Furthermore, 10 μM HCQ +30 μM AZM had a significantly larger effect in comparison to 3 μM HCQ +10 μM AZM (Figure 4E). We also assessed the time-dependent effect of combination therapy on contractility over the 5 min exposure period. As shown in Figure 4F, the lower concentration resulted in a significant reduction in contractility at ≥3 min superfusion, while the higher concentration led to a rapid significant reduction within the first minute of superfusion, which was sustained over the entire exposure period. HCQ and AZM combination also affected contraction kinetics in a concentration-dependent manner, with maximum slope of contraction significantly decreased at all concentrations (Figure 4G), although maximum slope of relaxation was significantly decreased only at 10 μM HCQ +30 μM AZM (Figure 4H).

### 3.5 The calcium channel agonist Bay K8644 prevents and reverses the effect of HCQ and AZM on contractility of porcine heart slices

**FIGURE 5:**
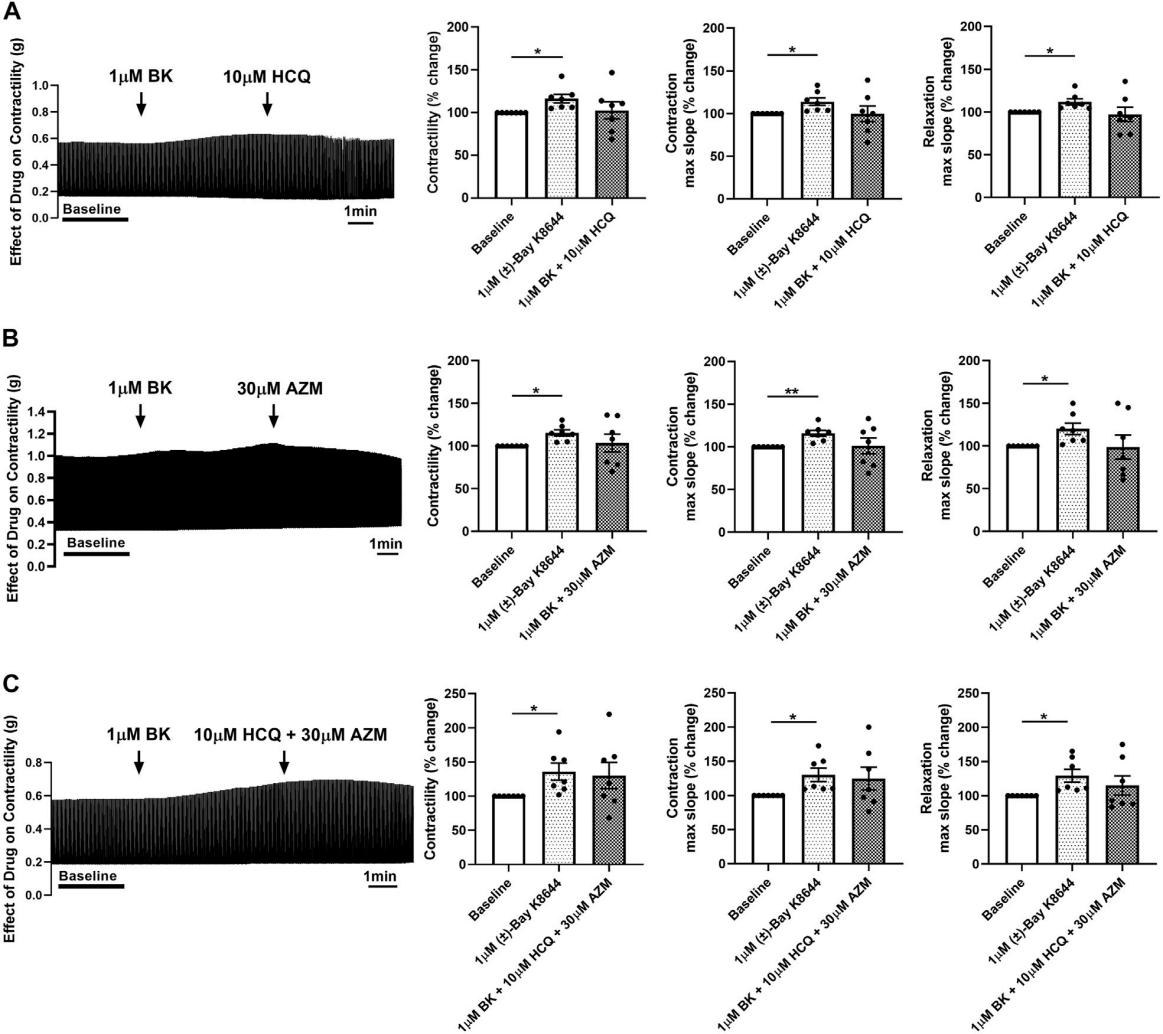
Pre-treatment of porcine heart slices with Bay K8644 prevents the effect of HCQ and AZM on cardiac contractility. **(A)** Representative contraction traces and bar graphs showing the effect of 1 μM (±)-Bay K8644 (BK) followed by 10 μM HCQ on contractile force, maximum slope of contraction and relaxation of porcine heart slices. **(B)** Representative contraction traces and bar graphs showing the effect of 1 μM BK followed by 30 μM AZM on contractile force, maximum slope of contraction and relaxation of porcine heart slices. **(C)** Representative contraction traces and bar graphs showing the effect of 1 μM BK followed by 10 μM HCQ + 30 μM AZM on contractile force, maximum slope of contraction and relaxation of porcine heart slices. (*n* = 7 slices/4 hearts; means ± SEM). *p < 0.05; **p < 0.01 (one-way ANOVA with Tukey’s multiple comparisons test).

**FIGURE 6:**
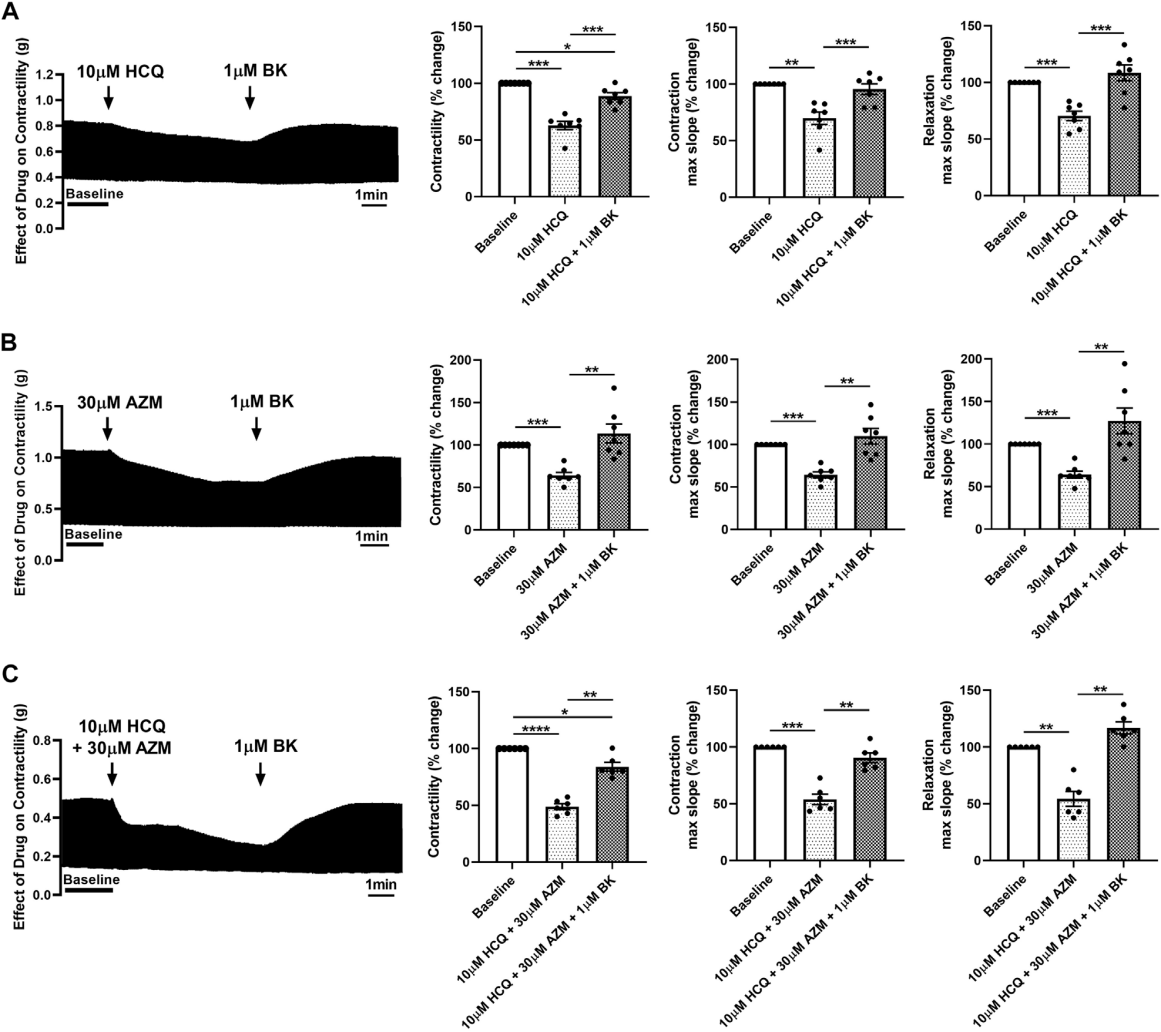
Bay K8644 reverses the effect of HCQ and AZM on contractility of porcine heart slices. **(A)** Representative contraction traces and bar graphs showing the effect of 1 μM (±)-Bay K8644 (BK) administered after 10 μM HCQ treatment on contractile force, maximum slope of contraction and relaxation of porcine heart slices. (*n* = 7 slices/4 hearts; means ± SEM). **(B)** Representative contraction traces and bar graphs showing the effect of 1 μM BK following 30 μM AZM on contractile force, maximum slope of contraction and relaxation of porcine heart slices. (*n* = 7 slices/4 hearts; means ± SEM). **(C)** Representative contraction traces and bar graphs showing the effect of 1 μM BK following 10 μM HCQ + 30 μM AZM on contractile force, maximum slope of contraction and relaxation of porcine heart slices. (*n* = 6 slices/4 hearts; means ± SEM). *p < 0.05; **p < 0.01, ***p < 0.001, ****p < 0.0001 (one-way ANOVA with Tukey’s multiple comparisons test).

To evaluate the potential role of L-type calcium channels in the reduction in contractility observed following treatment with HCQ and AZM, we assessed the effect of the two drugs individually and in combination after application of the calcium channel agonist Bay K8644. As shown in Figure 5, 1 µM Bay K8644 significantly increased contractility and maximum slope of contraction and relaxation when compared to baseline (Tyrode’s solution with 0.01% DMSO). Addition of 10 µM HCQ, 30 µM AZM or 10 µM HCQ +30 µM AZM had no effect on contractility, maximum slope of contraction or maximum slope of relaxation (Figure 5), suggesting activation of L-type calcium channels by Bay K8644 counteracts the effects of HCQ and AZM. Furthermore, we assessed the ability of Bay K8644 to reverse the effect of HCQ and AZM. As shown in Figure 6, 1 µM Bay K8644 completely reversed the effect of 10 µM HCQ, 30 µM AZM and 10 µM HCQ + 30 µM AZM on maximum slope of contraction and relaxation. Bay K8644 also completely reversed the effect of 30 µM AZM and significantly reduced the effect of 10 µM HCQ and 10 µM HCQ + 30 µM AZM on the contractile force of porcine slices. Taken together, these results suggest that L-type calcium channels are involved in the negative ionotropic effect of HCQ and AZM observed in porcine heart slices.

## 4 Discussion

In this study we explored the effect of HCQ and AZM applied alone or in combination on cardiac contractility by measuring contractile force and contraction kinetics in porcine ventricular tissue slices. The main findings can be summarized as follows: 1) HCQ monotherapy (1–10 µM) reduced contractile force and contraction kinetics in slices in a dose-dependent manner; 2) AZM monotherapy decreased contractile force and contraction kinetics only at higher concentrations (30 µM); 3) combination of HCQ and AZM induced a dose-dependent effect similar to HCQ alone; 4) pre-treatment of porcine slices with the L-type calcium channel agonist Bay K8644 prevented the effect of both drugs; 5) post-treatment of porcine slices with Bay K8644 largely reversed the effect of both drugs.

Our results show that treating porcine heart slices with increasing concentrations of HCQ alone induces contractile dysfunction, with both reduction in contractile force and decrease in contraction and relaxation velocity. HCQ has been recently reported to decrease contractile activity (sarcomere shortening) in single adult human cardiomyocytes in a dose-dependent manner (Jordaan et al., 2021), however no effect on contractile force was found in stem cell-derived cardiac tissue constructs treated with 1–10 µM HCQ (Wong et al., 2021). In our study we observed significant decrease in contractile force at ≥3 µM HCQ; this concentration is higher than that reported to induce significant decrease in sarcomere shortening in single human cardiomyocytes, where 1 µM HCQ induced a 50% reduction in sarcomere shortening (Jordaan et al., 2021). This may be due to differences in drug diffusion and sensitivity between single cells and intact myocardium, or different endpoints used to measure contractile activity (we measured contractile force directly while Jordaan et al. measured sarcomere shortening), although different effects due to species differences (human *versus* pig) cannot be excluded. Previous research has focused on chloroquine rather than its analogue HCQ. Depressed contractility and impairment in cardiac relaxation were observed in rats injected with 50 mg/kg/day chloroquine for 2 weeks (Chaanine et al., 2015). Decrease in contractile force and rates of contraction and relaxation were reported in rat left ventricular papillary muscles exposed to 100 µM chloroquine acutely (Riou et al., 1989), although the same chloroquine concentration had no effect on rat right ventricular papillary muscles in a different study (Hughes, 2020). In Langendorff-perfused guinea-pig and rat hearts however lower concentrations of chloroquine (>1 μM and >10 μM, respectively) administered acutely were sufficient to significantly decrease contractile function (Tona et al., 1990; Blignaut et al., 2019).

In our experiments, we chose intermediate concentrations at the clinically relevant levels, i.e., HCQ at 3 µM and AZM at 10 µM and extended the range to both lower and higher ends to assess the effects on organotypic heart slices. The blood concentrations at 1–2 μg/mL (2.3–4.6 µM) for HCQ and tissue content at 9.13–17.85 μg/g (concentrations 12–24 µM, taking specific density for tissue as 1) for AZM have been reported in patients receiving the treatments (Di Paolo et al., 2002; Ippolito and Flexner, 2020). Thus, our findings are of reference value to clinical applications.

In this study we show that treating porcine heart slices with AZM alone has no effect on slice contractile properties at 3 µM and 10 µM concentrations, however a higher concentration of 30 µM has a negative impact on contractile function, with reduced contractile force and slower contraction and relaxation kinetics. A similar reduction in contractile force and contraction/relaxation kinetics has recently been reported in stem cell-derived cardiac tissue constructs at ≥30 μM AZM (Wong et al., 2021). Similarly, no changes in contractile activity were observed in single human cardiomyocytes at ≤10 μM AZM (Jordaan et al., 2021), however the response to higher concentrations in human cardiomyocytes remains untested. In Langendorff-perfused rat hearts, acute administration of AZM (1–300 μM) decreased contractile force in a concentration-dependent manner, with an estimated half-maximal inhibitory concentration [IC50] of 51.9 ± 14 μM (Galan et al., 2017). This is in agreement with our data that show a reduction in contractile force of ∼42% after acute administration of 30 μM AZM. In halothane-anesthetized dogs, however, low doses of AZM (3 mg/kg, intravenously administered over 10 min) had no effect on ventricular contraction, and a significant negative inotropic effect was only detected at high doses (30 mg/kg) (Ohara et al., 2015). The above doses corresponded to a peak plasma concentration of 26 μM and 87 μM AZM, respectively.

Our results show that combined application of HCQ and AZM has similar effects to HCQ alone, reducing contractile force and decreasing contraction and relaxation velocities in a concentration-dependent fashion in porcine heart slices. Combination of HCQ and AZM has recently been reported to reduce contractile force in human stem cell-derived cardiac tissue constructs in a similar concentration-dependent manner (Wong et al., 2021). We report a 42% reduction in contractile force after treatment of slices with 10 μM HCQ +30 μM AZM, which is in line with the ∼40% reduction observed in human cardiac tissue constructs at the same drugs concentration (Wong et al., 2021). Combination of 10 µM AZM with 3 µM HCQ significantly reduced contractile function in our study, an effect we did not observe in slices treated with 10 µM AZM alone. HCQ has previously been reported to aggravate the negative inotropic effect of AZM in human heart tissue constructs (Wong et al., 2021). More pronounced contractile dysfunction was recently observed in human-induced pluripotent stem cell-derived cardiomyocytes treated with combined AZM and HCQ for 7 days, compared to respective mono-treatments (Li et al., 2022). In contrast, combination of HCQ and AZM in single human cardiomyocytes abolished the negative inotropic effect of HCQ monotherapy (Jordaan et al., 2021), although the study measured sarcomere shortening instead of contractile force. The significance of our findings is that these combination therapy effects are observed in native cardiac tissue with relevant mechanical and physiological properties.

Our data show that pretreating porcine heart slices with the L-type calcium channel agonist Bay K8644 prevents the effect of HCQ and AZM monotherapy/polytherapy on contractile force and contraction kinetics. Furthermore, application of Bay K8644 after treatment of slices with HCQ and AZM monotherapy/polytherapy largely reverses the effect of both drugs. This suggests that inhibition of L-type calcium channels, which play a key role in myocardial intracellular calcium rise and contractility (Eisner et al., 2017), may be the mechanism underlying the negative inotropic effect and contractile dysfunction produced by HCQ and AZM. Recent work using recombinant HEK-293 cells expressing the human Cav1.2 channel, reports HCQ inhibits the Cav1.2 channel with an IC50 of 7.64 µM (Jordaan et al., 2021), a concentration that generated significant contractile dysfunction in our study. Furthermore, HCQ has been reported to significantly reduce L-type calcium current in isolated guinea-pig sinoatrial node myocytes at 3 µM concentration (Capel et al., 2015). In contrast, another study on cell lines expressing Cav1.2 reports a lower effect of µM-HCQ concentrations on L-type calcium current, with an IC50 of 209 ± 94 µM (Wang et al., 2021). Chloroquine has also been found to affect L-type calcium current, with 10 μM chloroquine decreasing peak current by 32% ± 11% in isolated feline ventricular cardiomyocytes (Sanchez-Chapula et al., 2001). These studies together with previous work showing that the negative ionotropic effect of chloroquine can be antagonized by increasing extracellular calcium concentration (Ikhinmwin et al., 1981; Tona et al., 1990) support our conclusion that inhibition of L-type calcium channels plays an important role in the contractile dysfunction of porcine heart slices.

AZM has also been reported to affect L-type calcium current, although at higher concentrations. AZM was found to suppress L-type calcium current with an IC50 of 67 ± 4 µM in rabbit ventricular cardiomyocytes (Yang et al., 2017), and an IC50 of ∼1.2 mM in guinea-pig ventricular cardiomyocytes (Zhanget al. 2017). In recombinant HEK-293 cells expressing the human Cav1.2 channel, AZM was found to inhibit L-type calcium current with an IC50 of >300 µM and an IC50 ranging between 771 and 1391 µM in two independent studies (Delaunois et al., 2021; Jordaan et al., 2021). This inhibition could in part explain the contractile dysfunction observed in our porcine heart slices treated with AZM, an effect that was antagonized in the presence of the L-type calcium channel agonist Bay K8644.

Given the role that L-type calcium current plays in excitation-contraction coupling, as major trigger for calcium-induced calcium release from the sarcoplasmic reticulum, a decrease in calcium influx via L-type calcium channels could significantly affect intracellular calcium concentration and impair cardiac contraction (Eisner et al., 2017). The recent observation that HCQ decreases total calcium influx and calcium transient magnitude in cardiomyocytes (Oguntuyo et al., 2022; Segal et al., 2022), together with HCQ and AZM effect on calcium transient upstroke velocity in human induced pluripotent stem cell-derived cardiomyocytes (Charrez et al., 2021), further supports the ability of both drugs to reduce intracellular calcium concentration and affect contractility.

HCQ and AZM can also act on several different ion channels and pumps in addition to L-type calcium channels. Indeed, both drugs have been found to have some inhibitory effect on hERG, Na_V_1.5 and Kir2.1-mediated currents (Yang et al., 2017; Zhang et al., 2017; Delaunois et al., 2021; Jordaan et al., 2021; Wang et al., 2021). Furthermore, both drugs have been recently shown to affect cell viability, sarcomere organization, sarcomere length and mitochondrial function, all critical determinants of cardiac contraction (Chaanine et al., 2015; Jiang et al., 2019; Kim et al., 2021; Li et al., 2022). Further studies are thus needed to elucidate the contribution of ion channels, calcium signaling, structural disarrangement, and mitochondria dysfunction to the adverse effects of HCQ and AZM on cardiac contractile function.

In conclusion, our study is the first to assess the effect of HCQ and AZM on cardiac contractility in organotypic heart slices, an intact multicellular preparation which retains mechanical and physiological properties of native myocardium. Our findings reveal a negative effect of HCQ and AZM administered alone or in combination on cardiac contractile function and suggest a mechanism involving inhibition of L-type calcium channels. As preclinical safety testing focuses on QT interval prolongation and proarrhythmic risk, our study demonstrates the importance and feasibility of exploring other cardiotoxicity mechanisms. Finally, this study highlights the translational value of porcine heart slices as a human-relevant platform suitable for investigating mechanisms of drug cardiotoxicity.

## Data Availability

The raw data supporting the conclusion of this article will be made available by the authors, without undue reservation.
